# Boulder concentration effects on sediment transport and deposition

**DOI:** 10.1038/s41598-026-38978-7

**Published:** 2026-02-10

**Authors:** Penghua Teng, Dan A. Nilsson, Anders G. Andersson, J. Gunnar I Hellström

**Affiliations:** Division of Fluid and Experimental Mechanics, Department of Engineering Sciences and Mathematics, Luleå University of Technology, Luleå, SE-971 87 Sweden

**Keywords:** Boulder concentration, Turbulent flow, Sediment transport, Sediment deposition, Coupled CFD-DEM, Civil engineering, Fluid dynamics

## Abstract

Boulders in riverbeds significantly influence sediment transport and deposition by altering local flow patterns. This study employs coupled Computational Fluid Dynamics–Discrete Element Method (CFD–DEM) simulations to examine how boulder concentration affects sediment dynamics. Three boulder spacing scenarios, representing isolated, wake-interference, and skimming flow regimes, are evaluated for their impacts on flow structure, bed shear stress distribution, and sediment transport and deposition. The fluid phase is modelled using a large eddy simulation in a finite volume framework, while individual sediment grains are tracked with the discrete element method. The results reveal that increasing boulder concentration transforms the flow regime from isolated wakes behind individual boulders to a more coherent recirculation zone among neighboring boulders. This transition substantially reduces near-bed shear stresses between boulders and leads to a decline in total sediment transport rates. At high boulder concentration, sediment particles preferentially accumulate in sheltered inter-boulder corridors, forming stable depositional belts. In contrast, widely spaced boulders lead to isolated, localized deposition around individual boulders. To our knowledge, this is the first attempt to apply a coupled finite volume–DEM approach to simulate boulder–sediment interactions in open-channel flow, enabling analysis of sediment transport and deposition under varying boulder concentrations.

## Introduction

Large roughness elements in rivers are commonly arranged in arrays to support stream restoration efforts^1^. By altering the surrounding flow field, these large roughness elements can improve both the availability and quality of aquatic habitats^2,3^. Large, immobile boulders are usually treated as a large-scale roughness in natural gravel-bed rivers as the ratio between flow water depth *H* and the boulder diameter *d*_*84*_, which is the diameter for which 84% of the particles are finer, is *H*/*d*_*84*_ ≤4 ^4–7^. The presence of the boulders in riverbeds substantially impacts these processes by modifying local hydrodynamic conditions, inducing complex flow separation, turbulence, and characteristic wake structures^8,9^. Consequently, these flow alterations significantly influence sediment entrainment, transport dynamics, and deposition patterns^10–12^.

Boulders in riverbeds can be arranged in various configurations, ranging from isolated individual elements to dense clusters. These different configurations induce distinct flow regimes: isolated roughness flow, wake interference flow, and skimming flow, categorized mainly according to the spacing between boulders^13–17^. For instance, flume experiments by Papanicolaou et al.^14^ identified these regimes using boulder spacings of 6, 2, and 1 times the boulder diameter, respectively. In these regimes, complex flow structures such as recirculation zones, horseshoe vortices, and turbulence wake structures are prevalent^12,18–22^. These coherent structures intensify local turbulence, influence shear stress distribution, and significantly affect vertical turbulence profiles^17,20^.

The influence of boulders on bedload transport has been explored primarily through flume experiments and numerical modelling, typically focusing on how boulders modify flow resistance^15,18,23–25^. Flow resistance, often quantified via shear stress, has been shown to depend significantly on boulder concentration. In isolated roughness conditions, flow resistance typically increases proportionally with boulder concentration, whereas in wake interference and skimming flows, this relationship is more complex and nonlinear^16,25^. Numerical simulations by Fang et al.^15^ also demonstrated that increasing boulder concentration enhances shear stress variability, influencing sediment transport processes.

Despite these insights, the complex interactions among flowing water, boulders, and sediment particles have not yet been fully described or visualized. Few studies have specifically observed or quantified how variations in boulder spacing influence bedload transport and deposition. Notably, experimental studies by Papanicolaou et al.^16–27^ revealed that isolated boulder arrays significantly reduce bedload transport, creating upstream sand ridges and stoss-side depositional patches. These observations further illustrate the significant influence of boulders on sediment transport and deposition processes, emphasizing the need for detailed investigations to quantify and visualize the processes, especially in applications related to river restoration and ecological habitat design.

To address these knowledge gaps, numerical modelling has emerged as alternative tool for simulating sediment transport in boulder-rich river channels. In recent decades, various numerical methods have been successfully used to simulate particle-fluid flows, with the coupled lattice Boltzmann method (LBM)-discrete element model (DEM) and computational fluid dynamics (CFD)-DEM being the most applied in engineering fields such as hydraulic fracturing, coastal sediment transport, gas-solid fluidization, and aerosol deposition. Additionally, Robinson et al.^28^ used a coupled Smoothed Particle Hydrodynamics (SPH)-DEM model to simulate single and multiple particle sedimentation in a three-dimensional (3D) fluid column.

The current study employs a coupled CFD-DEM approach to simulate sediment transport and deposition under varying boulder concentrations, which allows systematic variation of boulder concentration under controlled boundary conditions and provides full access to velocity, shear stress, and particle dynamics that are difficult to obtain experimentally. In this method, the Navier-Stokes equations are solved using the finite volume method (FVM) to describe fluid motion, while individual particle movement is modelled using DEM based on Newton’s laws. The CFD-DEM approach has been successfully applied to particle-laden flows such as sediment transport, geodynamical magmatic flows, seepage flows and rock erosion^29–34^. This approach effectively captures the dynamic interaction between flowing water and sediment particles, making it particularly suited for studying the influence of boulder concentration on sediment transport and deposition.

This study investigates sediment transport and deposition patterns across three boulder spacing scenarios, corresponding to the isolated (10 times boulder width), wake interference (5 times boulder width), and skimming (2 times boulder width) flow regimes. By systematically comparing these cases, the study quantifies changes in flow structures, sediment transport pathways and deposition patterns. Insights from the study are expected to improve the understanding of sediment dynamics in boulder-rich rivers and provide practical insights for restoration efforts aimed at rehabilitating degraded waterways and enhancing aquatic habitats.

## Methodology

### Coupled CFD-DEM framework

This study employs a coupled CFD-DEM approach to investigate sediment transport and deposition under different boulder spacings. The coupled CFD-DEM approach integrates two open-source software packages: OpenFOAM and LIGGGHTS. In this framework, CFD solves the Navier-Stokes equations to describe fluid behavior, while DEM simulates particle dynamics based on Newton’s laws, treating sediment as discrete particles. The CFD-DEM engine enables coupling between fluid and particles, as described in the section of CFD-DEM Coupling Process. Further details on the software can be found in Kloss et al.^35^ and Goniva et al.^36^

### Fluid motion model

In this study, fluid motion is modelled using Large Eddy Simulation (LES), where the spatially filtered 3D Navier-Stokes equations are solved over time. This approach directly resolves eddy motions larger than the numerical grid mesh size, while smaller-scale motions are represented using a sub-grid scale model.

The fluid momentum equation is formulated as

1 $$\:{\rho\:}_{f}\left(\frac{\partial\:{\epsilon\:}_{f}{\boldsymbol{v}}_{\boldsymbol{f}}}{\partial\:t}+\nabla\:\cdot\:\left({\epsilon\:}_{f}{\boldsymbol{v}}_{\boldsymbol{f}}{\boldsymbol{v}}_{\boldsymbol{f}}\right)\right)=-\nabla\:p-{\boldsymbol{f}}_{\boldsymbol{f}}^{\boldsymbol{p}}+\nabla\:\cdot\:\tau\:+{\rho\:}_{f}{\epsilon\:}_{f}\boldsymbol{g}$$

where $\:{\rho\:}_{f}$ is the fluid density (kg/m³), $\:{\boldsymbol{v}}_{\boldsymbol{f}}$ is the fluid velocity (m/s), and ***g*** denotes gravitational acceleration (m/s²). $\:{\epsilon\:}_{f}$ refers to the fluid volume fraction within a cell, while $\:\nabla\:p$ is the pressure gradient. The term $\:{\boldsymbol{f}}_{\boldsymbol{f}}^{\boldsymbol{p}}=\sum\:_{i=1}^{Np}{\boldsymbol{f}}_{\boldsymbol{p}\boldsymbol{i}}^{\boldsymbol{f}}/\varDelta\:V$ denotes the force per unit volume exerted by particles on the fluid, where $\:\varDelta\:V$ is the mesh-cell volume (m³), *N*_*p*_ is the number of particles within the cell, and $\:{\boldsymbol{f}}_{\boldsymbol{p}\boldsymbol{i}}^{\boldsymbol{f}}$ is the interaction force exerted by the fluid on the *i*th particle in the cell (N).

The unresolved subgrid fluid stress, $\:\tau\:=-2{\nu\:}_{t}\stackrel{-}{{S}_{ij}}$, is provided by the dynamic Smagorinsky model^37^, where $\:\stackrel{-}{{S}_{ij}}$ represents the resolved strain rate (s^− 1^), and $\:{\nu\:}_{t}={\left({C}_{s}{\Delta\:}\right)}^{2}\sqrt{2\stackrel{-}{{S}_{ij}}\stackrel{-}{{S}_{ij}}}$ is the sub-grid scale eddy viscosity (m²·s⁻¹). Here, $\:{\Delta\:}$ is the characteristic filter length (m), defined as the cubic root of the cell volume, and $\:{C}_{s}$ is the Smagorinsky constant, dynamically adjusted to local flow conditions. The LES model has been widely applied to simulate flow around bluff bodies^38–41^.

### Particle motion model

The DEM is a Lagrangian approach used to compute the dynamics of individual particles. Newton’s second law governs the force and torque equations, which are expressed as follows:

2 $$\:{m}_{p}\frac{d{\boldsymbol{v}}_{\boldsymbol{p}}}{dt}={\boldsymbol{f}}_{\boldsymbol{p}}^{\boldsymbol{g}}+{\boldsymbol{f}}_{\boldsymbol{p}}^{\boldsymbol{c}}+{\boldsymbol{f}}_{\boldsymbol{p}}^{\boldsymbol{f}}$$

3 $$\:{I}_{p}\frac{{dw}_{p}}{dt}={T}_{P}^{c}+{T}_{p}^{f}$$

where $\:{\boldsymbol{v}}_{\boldsymbol{p}}\:$ and $\:{\boldsymbol{\omega\:}}_{\boldsymbol{p}}$are the translational velocity (m/s) and angular velocity (rad/s) of an individual particle, respectively. $\:{\boldsymbol{f}}_{\boldsymbol{p}}^{\boldsymbol{g}}={m}_{p}g$ denotes the gravitational force (N), where $\:{m}_{p}$ is the particle mass (kg). $\:{\boldsymbol{f}}_{\boldsymbol{p}}^{\boldsymbol{c}}\:$ is the contact force (N) resulting from interparticle collisions, while $\:{\boldsymbol{f}}_{\boldsymbol{p}}^{\boldsymbol{f}}\:$ is the particle-fluid interaction force (N) exerted by the surrounding fluid (introduced in Sect. 2.1.3). The angular moment of inertia is given by $\:{I}_{p}\:$ (kg·m²), and $\:{T}_{p}^{c}$ is the torques (kg·m²/s²) generated by particle-particle or particle-wall collisions, modelled using an elastic spring and a viscous damper. Further details can be found in Tsuji et al.^42^. Additionally, $\:{T}_{p}^{f}$ denotes the torques (kg·m²/s²) caused by particle-fluid interaction forces acting on the particle’s centroid.

### Contact forces

The contact force model, based on the work of Mindlin and Deresiewicz^43^, employs Hertzian contact theory and the tangential force-displacement relationship. In this model, particles are treated as elastic spheres, and the$\:{\boldsymbol{f}}_{\boldsymbol{p}}^{\boldsymbol{c}}\:$ due to particle-particle or particle-wall collisions is defined by normal$\:{\boldsymbol{f}}_{\boldsymbol{n}}\:$ and tangential$\:{\boldsymbol{f}}_{\boldsymbol{t}}\:$ forces (N), expressed as follows:

4 $$\:{\boldsymbol{f}}_{\boldsymbol{p}}^{\boldsymbol{c}}={\boldsymbol{f}}_{\boldsymbol{n}}+{\boldsymbol{f}}_{\boldsymbol{t}}=\left({k}_{n}{\delta\:}_{n}-{r}_{n}{\boldsymbol{v}}_{\boldsymbol{n}}\right)+\left({k}_{t}{\delta\:}_{t}-{r}_{t}{\boldsymbol{v}}_{\boldsymbol{t}}\right)\:\:$$

where $\:{\boldsymbol{v}}_{\boldsymbol{n}}$ and $\:{\boldsymbol{v}}_{\boldsymbol{t}}$ are the relative velocities (m/s) of two particles in the normal and tangential directions, respectively, while $\:{\delta\:}_{n}$ and $\:{\delta\:}_{t}$ denote their overlap distances (m) in these directions. The tangential force is a “history” effect that accounts for the tangential displacement ($\:{\delta\:}_{t}$) between the particles for the duration of the time they are in contact. The $\:{\delta\:}_{t}$ is calculated by integrating the relative tangential velocity at the contact point over time. The coefficient of friction, , is the upper limit of $\:{\boldsymbol{f}}_{\boldsymbol{t}}$ through the Coulomb criterion. Thus, in the Hookean case, the tangential force between 2 particles grows according to a tangential spring and dash-pot model until $\:{\boldsymbol{f}}_{\boldsymbol{t}}$/$\:{\boldsymbol{f}}_{\boldsymbol{n}}$ = and is then held at $\:{\boldsymbol{f}}_{\boldsymbol{t}}$= $\:{\boldsymbol{f}}_{\boldsymbol{n}}$ until the particles lose contact.

The elastic stiffness of a particle (N/m) in the normal and tangential directions is given by:

5 $$\:{k}_{n}=\frac{4}{3}{E}^{*}\sqrt{{R}^{*}{\delta\:}_{n}}$$

6 $$\:{k}_{t}=8{G}^{*}\sqrt{{R}^{*}{\delta\:}_{n}}$$

where $\:{R}^{*}=\frac{1}{{R}_{i}}+\frac{1}{{R}_{j}}$, $\:{E}^{*}=\frac{{-\vartheta\:}_{i}^{2}+1}{{E}_{i}}+\frac{{-\vartheta\:}_{j}^{2}+1}{{E}_{j}}$ and $\:{G}^{*}=\frac{2(2+{\vartheta\:}_{i})}{{E}_{i}}+\frac{2(2+{\vartheta\:}_{j})}{{E}_{j}}$. *R*, *E*, and $\:\vartheta\:$ are the radius, the Young’s modulus and the Poisson’s ratios of two contacting particles *i* and *j*. The viscoelastic damping constants (kg/s) of normal and tangential contact are

7 $$\:{r}_{n}=-2\sqrt{5/6}\beta\:\sqrt{{s}_{n}{m}^{*}}$$

8 $$\:{r}_{t}=-2\sqrt{5/6}\beta\:\sqrt{{s}_{t}{m}^{*}}$$

where $\:{S}_{n}=2{E}^{*}\sqrt{{R}^{*}{\delta\:}_{n}}$, $\:{S}_{t}=8{G}^{*}\sqrt{{R}^{*}{\delta\:}_{n}}$, $\:{m}^{*}=\frac{1}{{m}_{pi}}+\frac{1}{{m}_{pj}}$ and $\:\beta\:=\frac{\mathrm{l}\mathrm{n}\left(e\right)}{\sqrt{{(\mathrm{l}\mathrm{n}(e\left)\right)}^{2}+{\pi\:}^{2}}}$ in which e is the coefficient of restitution.

Also, the Elastic-Plastic Spring-Dashpot (EPSD) model is employed to describe rolling resistance between contacting particles in the present study. The rotation torque in the model consists of two components: a mechanical spring and viscous damping torques. The total rolling fraction torque is written as:

9 $$\:{M}_{r}={M}_{r}^{k}+{M}_{r}^{d}$$

where $\:{M}_{r}^{k}$ and $\:{M}_{r}^{d}$ (N·m) are the spring and viscous damp torques, respectively. More specific details of the model can be found in Ai et al.^49^ Model Type C.

### CFD-DEM coupling process

**Fig. 1:**
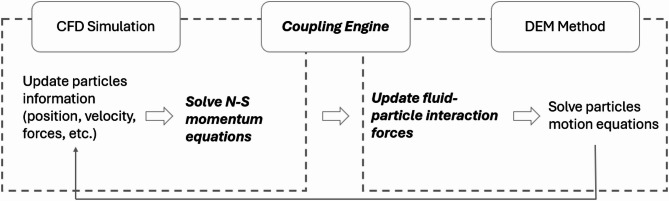
Flowchart of the CFDEM coupling process.

The CFD-DEM coupling process is shown in Fig. 1. The process begins with the initialization of particles in the DEM, where a contact model is employed to calculate particle contact forces and momentum. According to Newton’s second law, the motion of each particle is determined, and their velocities and positions are updated accordingly. The updated particle information is then transferred to the CFDEM coupling engine, which delivers the data to the CFD solver. Upon receiving this information, the CFD solver calculates the particle volume fraction and averaged mean velocity within each cell, and subsequently solves the Navier–Stokes equations to determine the fluid forces acting on the particles. These fluid forces are finally fed back into the DEM solver as interaction forces, which are used to further update the particles’ motion, thereby completing the coupling loop.

The coupling between CFD and DEM is unresolved because the particle-fluid interaction forces are calculated through force models rather than directly integrate forces acting on a particle’s surface, as described by Zhou et al.^44^. The forces acting on a particle are computed based on the particle volume fraction and mean particle velocity within a CFD cell. The formulation of the interaction force, $\:{\boldsymbol{f}}_{\boldsymbol{p}}^{\boldsymbol{f}}$, is typically problem-specific. This study aims to simulate the process of sediment transport and deposition. Therefore, $\:{\boldsymbol{f}}_{\boldsymbol{p}}^{\boldsymbol{f}}$ includes the fluid pressure gradient, buoyancy, viscous, drag, and lift forces, as these primarily govern particle-fluid interactions^35,44^. The interaction force $\:{\boldsymbol{f}}_{\boldsymbol{p}}^{\boldsymbol{f}}$ is then formulated as follows:

10 $$\:{\boldsymbol{f}}_{\boldsymbol{p}}^{\boldsymbol{f}}={\boldsymbol{f}}_{\boldsymbol{d}}+{\boldsymbol{f}}_{\boldsymbol{l}}+{\boldsymbol{f}}_{\nabla\:\boldsymbol{p}}+{\boldsymbol{f}}_{\boldsymbol{v}}$$

where the $\:{\boldsymbol{f}}_{\boldsymbol{d}}$, $\:{\boldsymbol{f}}_{\boldsymbol{l}}$, $\:{\boldsymbol{f}}_{\nabla\:\boldsymbol{p}}$ and $\:{\boldsymbol{f}}_{\boldsymbol{v}}$ are the drag, lift, pressure gradient including the effect of buoyancy and viscous forces, respectively.

The $\:{\boldsymbol{f}}_{\boldsymbol{d}}$ is defined by the following equation, as used by Schmeeckle^45^, who modelled bedload transport conditions:

11 $$\:{\boldsymbol{f}}_{\boldsymbol{d}}=\frac{1}{8}\pi\:{\rho\:}_{f}{C}_{d}{D}^{2}\left|{\boldsymbol{v}}_{\boldsymbol{p}}-{\boldsymbol{v}}_{\boldsymbol{f}}\right|({\boldsymbol{v}}_{\boldsymbol{p}}-{\boldsymbol{v}}_{\boldsymbol{f}})$$

where $\:{\boldsymbol{v}}_{\boldsymbol{p}}$ is the particle velocity vector (m/s), *D* is the particle diameter (m) and $\:{C}_{d}$ is the drag coefficient calculated as

12 $$\:{C}_{d}={(0.9+\frac{4.8}{\sqrt{{Re}_{r}}})}^{2}$$

where $\:{Re}_{r}$ is the relative Reynolds number

13 $$\:{Re}_{r}=\frac{\left|{\boldsymbol{v}}_{\boldsymbol{p}}-{\boldsymbol{v}}_{\boldsymbol{f}}\right|{\rho\:}_{f}D}{{\mu\:}_{f}}$$

where $\:{\mu\:}_{f}$ is the dynamic water viscosity (kg/(m∙s)).

The $\:{\boldsymbol{f}}_{\boldsymbol{l}}$ acting on a spherical particle is formulated according to Saffman lift arising from the pressure distribution on a particle in a velocity gradient^46^ as

14 $$\:{\boldsymbol{f}}_{\boldsymbol{l}}={C}_{l}{\rho\:}_{f}{\nu\:}^{0.5}{D}^{2}({\boldsymbol{v}}_{\boldsymbol{p}}-{\boldsymbol{v}}_{\boldsymbol{f}})\times\:\left|{\boldsymbol{v}}_{\boldsymbol{p}}-{\boldsymbol{v}}_{\boldsymbol{f}}\right|$$

where $\:\nu\:$ is the kinematic viscosity (m^2^∙s^− 1^) and $\:{C}_{l}=$ 1.6 is the lift coefficient. The viscous and pressure gradient forces are formulated as $\:{\boldsymbol{f}}_{\boldsymbol{v}}=-{v}_{p}\times\:\nabla\:\cdot\:\tau\:$ and $\:{\boldsymbol{f}}_{\nabla\:\boldsymbol{p}}=-{v}_{p}\times\:\nabla\:p$, respectively.

### Model validation

To validate the numerical model, sediment transport is simulated based on the study by Schmeeckle^45^. In these validation cases, a pressure gradient is applied to each unit volume to drive the flow, ensuring that the spatially averaged fluid velocity in the computational domain reaches a predefined target velocity. The target velocities (*v*_*target*_) range from 0.6 to 1.1 m/s in increments of 0.1 m/s.

**Fig. 2:**
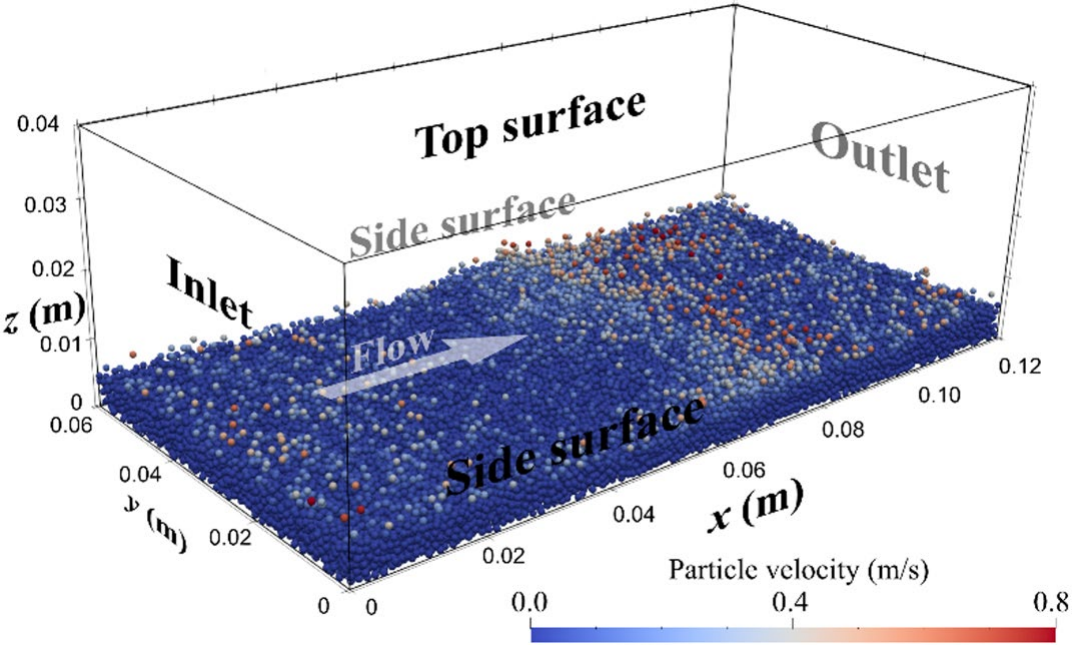
Computational domain and instantaneous velocity of particles under = 0.8 m/s.

Figure 2 illustrates the computational domain, where particles are coloured based on their velocity. The dimensions of the domain are 0.12 × 0.06 × 0.04 m, consisting of 468,000 hexahedral cells. A total of 60,000 particles are created to ensure the near-zero particle and fluid velocities in the vicinity of the bottom. The boundary conditions, shown in Fig. 2, include periodic boundaries at the inlet, outlet, and side surfaces, a rigid lid at the top, and a no-slip condition at the bottom. Table 1 lists the particle parameters, where the sand particles are represented as elastic spheres with a diameter of 0.001 m. Previous numerical studies have shown that reducing Young’s modulus (*E*) has minimal impact on particle behavior in DEM simulations^44,47^^48^. Therefore, to optimize computational efficiency by increasing the simulation time-step size, *E* is set to 5 × 10⁸ Pa, which is lower than its actual value. The rolling resistance coefficient is set to 0.3 since this value resulted in good performance in predicting the sandpile profile in Ai et al.^49^. All particle-particle collisions are expected to be viscously damped, and the coefficient of restitution is thus set to 0.01 based on the study of Schmeeckle^45^. Based on monitoring the interaction of a settling sphere with a wall, it is confirmed that particle collisions result in negligible rebound.

**Table 1:** Particle parameters.

Parameters	values
Particle diameter (*D*)	0.001 m
Coefficient of restitution (*e*)	0.01
Poisson’s ratio ($\:\vartheta\:$)	0.45
Young’s modulus (*E*)	5 × 10^8^ Pa
Coefficient of friction ()	0.6
Coefficient of rolling friction	0.3
Particle density ($\:{\rho\:}_{p}$)	2650 kg/m^3^

**Fig. 3:**
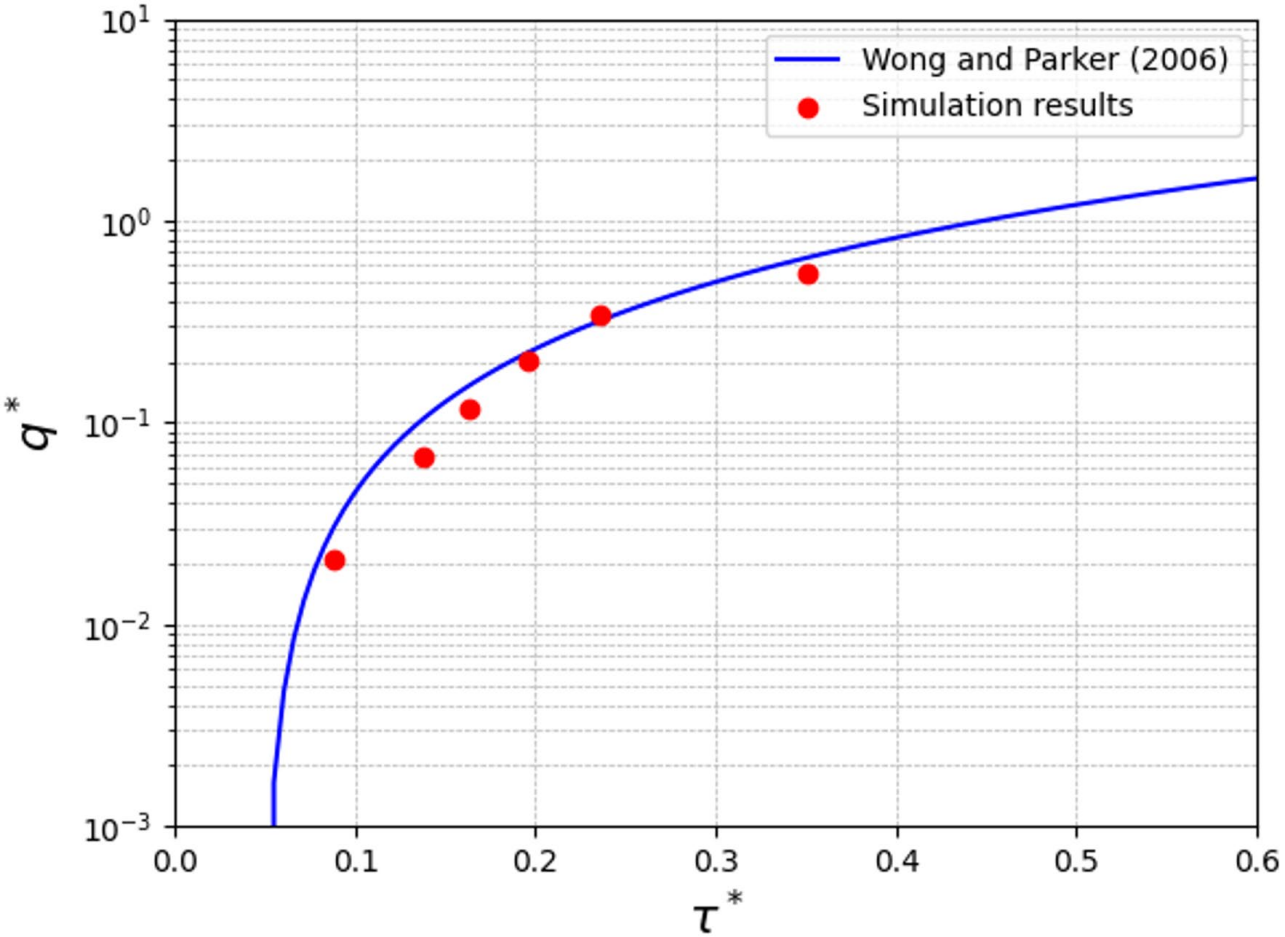
Comparison of the simulation results with the empirical equation, $\:{q}^{*}=\:3.97\cdot\:{({\tau\:}^{*}-0.0495)}^{1.5}$.

Figure 3 shows that the simulation results have a good agreement with the empirical bedload transport equation, $\:{q}^{*}=\:3.97\cdot\:{({\tau\:}^{*}-0.0495)}^{1.5}$, formulated by Wong and Parker^50^ derived from a reanalysis of the flume data of Meyer-Peter and Müller^59^, where$\:{\tau\:}^{*}$ is the Shields stress and calculated as $\:{\tau\:}^{*}={\tau\:}_{w}/({\rho\:}_{p}-{\rho\:}_{f})gD$, where the$\:{\tau\:}_{w}$ is the bed shear stress (Pa), and $\:{\rho\:}_{p}$ is the particle density (m^3^/s), and the Einstein number, $\:{q}^{*}$, is formulated as

15 $$\:{q}^{*}=\:{q}_{v}/{\left(\left(\frac{{\rho\:}_{p}}{{\rho\:}_{f}}-1\right)g{D}^{3}\right)}^{0.5}$$

where $\:{q}_{v}$ is the volumetric flux (m^3^/s) calculated as the time-averaged mean of all particles’ downstream velocity, multiplied by the total volume of particles, and divided by the area of the horizontal plane excluding the area occupied by boulders.

The results indicate that the numerical model effectively captures particle motion influenced by the surrounding water flow. Furthermore, previous studies^51,52^ have successfully validated the CFD-DEM approach for various particle-laden flows. These validations and applications provide evidence that the method reliably represents particle-fluid interactions.

### Simulation cases

#### Computational domain

**Fig. 4:**
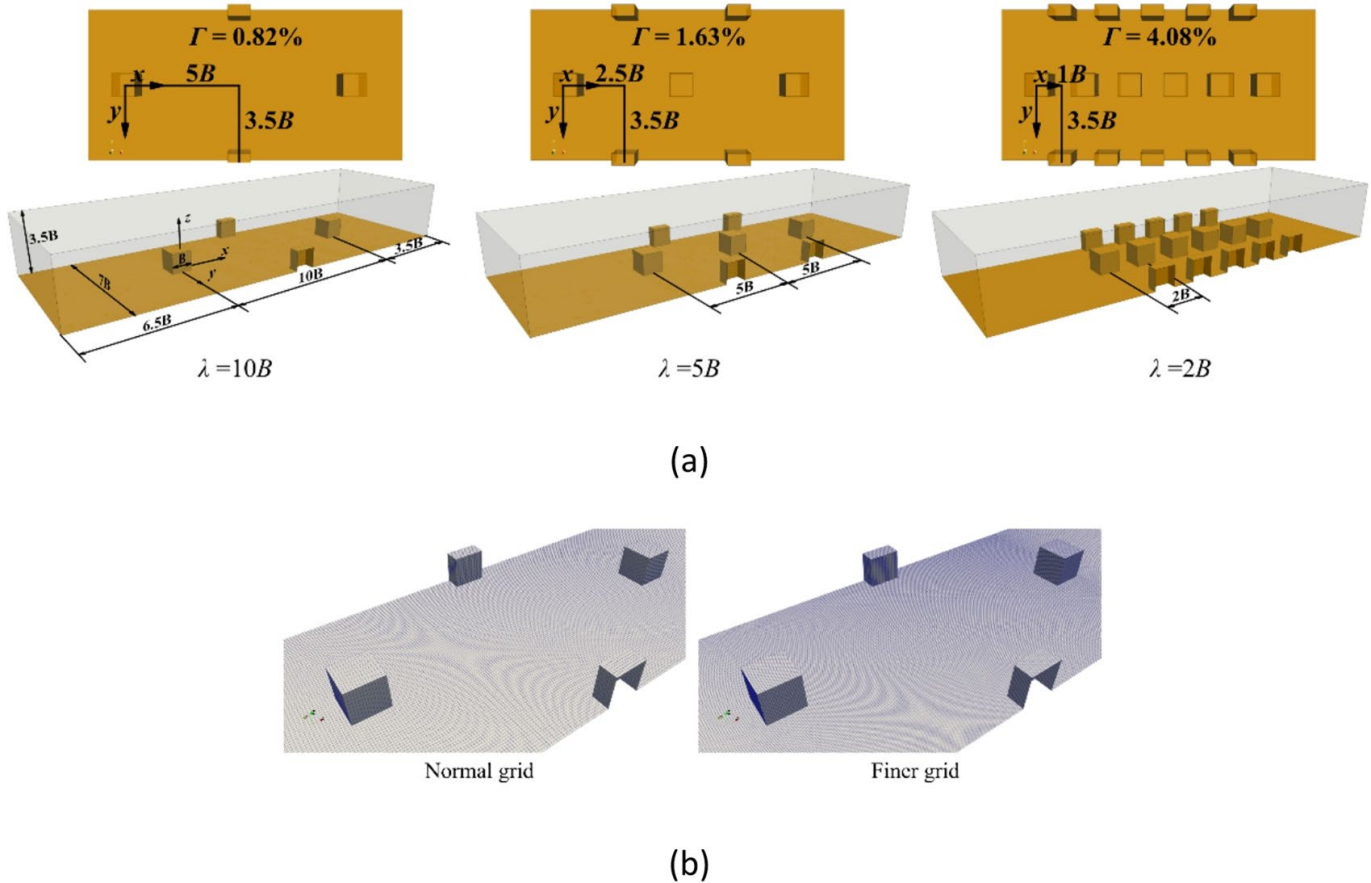
Computational domain (**a**) dimensions of simulations with *λ* = 10*B*, 5*B*, and 2*B*; (**b**) normal and finer grids of simulation *λ* = 10*B*.

Previous studies have employed hemispherical or spherical boulders to examine their influence on local hydrodynamic conditions, providing valuable insights for river restoration efforts^14–19^. Additionally, Hohermuth and Weitbrecht^53^ highlighted that angular, block-shaped boulders can improve hydraulic stability and habitat complexity. Accordingly, the present study adopts cube-shaped boulders with a fixed width of *B* = 50 mm. The effects of boulder concentration on sediment transport and deposition are investigated by varying the streamwise boulder spacing, $\:\lambda\:$, of staggered cube boulders. Three different values of $\:\lambda\:$, $\:\lambda\:$ = 2*B*, 6*B* and 10*B*, are selected based on the study of Fang et al.^15^. They used same $\:\lambda\:$ values to investigate the influence of boulder concentration on turbulence and sediment transport over submerged spherical boulders. Figure 4a shows the computational domain of three simulation cases. Each domain extends 20*B* in the streamwise direction, 7*B* in the spanwise direction, and 3.5*B* in the vertical direction. Boulders are located 6.5*B* downstream from the inlet and 3.5*B* upstream of the outlet. The value of $\:\lambda\:$ is varied among the three cases, ranging from 2*B* to 10*B*. A coordinate system is defined with the *x*- and *y*-axes lying on the channel bed, and the origin is located at the centre of the first boulder’s base.

The computational grids of the three cases contain 1 350 000, 980 000 and 860 000 hexahedral cells corresponding with $\:\lambda\:=$ 10*B*, 5*B* and 2*B*, respectively. The mesh is refined around the boulders to better capture the interaction between sediment, flowing water and the boulders. Since unresolved coupling CFD-DEM used in this study deals with particles’ size (introduced in the following section) smaller than the CFD computational grid, the smallest size of the grid cell, 2.6 mm × 2.4 mm × 2.6 mm, is close to the particle size to increase the accuracy of the fluid-filled void area. In addition, to study the grid independence of the simulation, a more refined grid of the large-spacing case ($\:\lambda\:=$ 10*B*) shown in Fig. 4b is also generated. The number of cells is 2 022 000, and the finer grid is generated around the boulders.

#### Fluid and particle parameters

As the submergence ratio has been shown to influence the flow pattern around boulders, the boulder concentrations, *Γ*, is defined as the volume of boulders per volume of fluid to include the vertical distribution of the boulders. The *Γ* values for the large-, medium-, and small-spacing simulations are 0.82%, 1.63% and 4.08% respectively. The flow of all simulations is turbulent with a Reynolds number of 52,500, $\:Re=H{v}_{bulk}/\nu\:$, where *H* is water depth (m), $\:{v}_{bulk}$ is bulk water velocity (m/s) with a value of 0.35 which is maintained constant by a pressure gradient in the computational domain, and $\:\nu\:$ is kinematic viscosity (m^2^ s^− 1^). Papanicolaou et al.^26^ used fine sand to investigate sediment transport and deposition under isolated boulder conditions in flume experiments. Additionally, Zheng et al.^53^ conducted a series of experiments to investigate the erosion of two different sizes of fine sand sediment by released flows. Accordingly, fine sand is selected as sediment particles in the present study and modelled as elastic spheres. The sand parameters used in the current model are listed in Table 1, but the value of *D* is 1.2 mm to reduce the number of particles for each case for saving computational cost.

#### Boundary conditions and initial scenarios

**Fig. 5:**
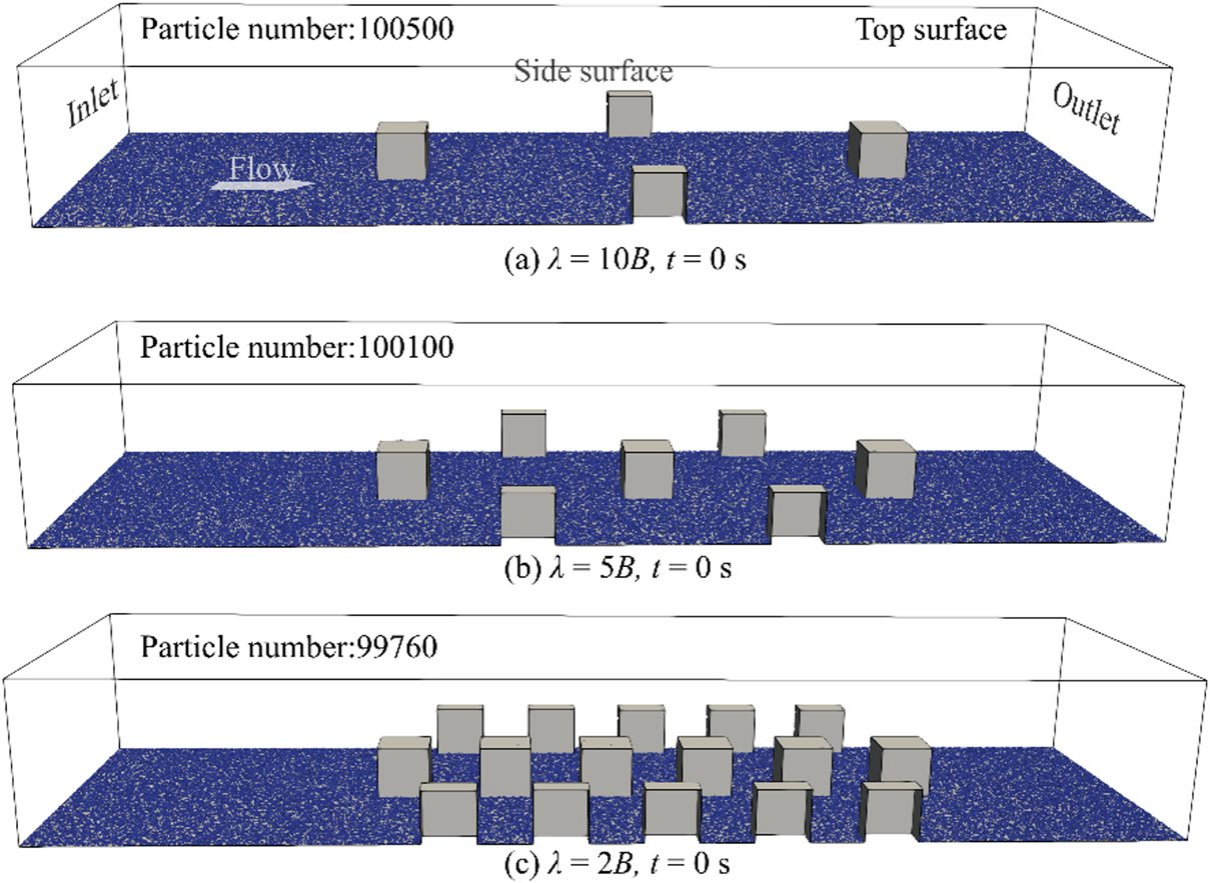
Boundary conditions and initial scenarios of simulations.

Boundary conditions for all simulations are shown in Fig. 5a. The inlet, outlet and side surfaces are set as periodic boundary conditions. The top and bottom surfaces are treated as slip wall and no-slip wall boundary conditions. Figure 5a-c show the initial conditions (simulation time, *t* = 0 s) of particles in the three simulation cases. To achieving the initial condition, in each case, particles are inserted from the top surface with a constant vertical velocity, and the bottom surface of the computational domain is then gradually covered by the uniform-sized particles (*D* = 1.2 mm). Finally, the particles attain a stationary state (*v*_*p*_ = 0 m/s). The particle numbers of large-, medium-, and small-spacing simulations are 100 500, 100 100 and 99 760, respectively. The number of particles in each case is kept approximately the same to investigate the effect of boulder concentration on sediment transport and to ensure sufficient particles to cover the bottom surface in each scenario.

## Results

### Time-Averaged flow

**Fig. 6:**
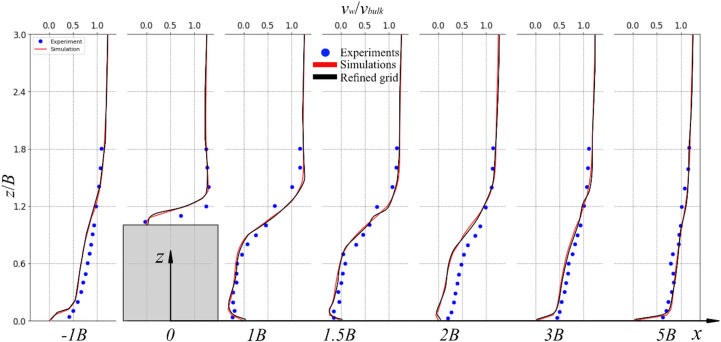
Profiles of the normalized streamwise velocity at the transect *y* = 0 for *λ* = 10*B*.

Since the large-spacing, *λ* = 10*B*, boulder case can be treated as an isolated regime^14^, the simulation results of that case are compared with the study by Singh et al.^55^. They conducted experiments to investigate the turbulence characteristics of flow over an isolated cube boulder (*B* = 50 mm), which matches the boulder size used in the present study. Figure 6 shows the simulated and experimental profiles of the time-averaged normalized streamwise velocity, *v*_*w*_/*v*_*bulk*_, at selected locations from − 1*B* to 5*B* at the transect *y* = 0, where the velocity measurements were carried out. Moreover, a grid comparison is also performed, indicating good agreement, compare the red and black curves in Fig. 6.

Because of the presence of the boulder at *x* = 0, the local flow field is altered, forming a recirculation zone downstream of the boulder. The time-averaged streamwise velocity below the boulder crest is decelerated both upstream and downstream of the boulder, especially near the bed. At the location *x* = -1*B*, the streamwise velocity near the bed is strongly decelerated in experiments and simulations. From 1*B* to 2*B*, the reverse flow is observed at *z*/*B* < 1, and in the simulation, at *x* = 2*B*, a recirculation region is still present near the bottom compared with the experiment. This discrepancy may be due to the presence of particles at the bed which influences the development of the recirculation region (Fig. 8). From 3*B* to 5*B*, the flow behaves more like the flow over a flat plate bed^54^, and the predicted profiles are generally in good agreement with the measured data. Comparisons of the numerical simulations with experimental data highlight the credibility of the LES for describing the flow features. Furthermore, as indicated in Fig. 6, the refined grid case shows good agreement with the results of the normal grid. This implies that the simulation results are independent of the numerical grid. The normal grid will be used in the medium- ($\:\lambda\:=$ 5*B*), and small-spacing ($\:\lambda\:=$ 2*B*) simulations.

### Local flow field

The presence of a boulder induces flow deceleration both upstream and downstream, recirculation and formation of a wake. The reduction in flow velocity associated with varying boulder concentrations is quantified using the streamwise velocity reduction, *Δv*, below *z/B* = 0.9, as suggested by Dey et al.^11^ and Fang et al.^15^, They defined the value of *Δv* as the difference in streamwise velocity between the non-boulder and the with-boulder conditions (*λ* = 2*B*, 5*B* and 10*B*). In this study, the streamwise velocity of the non-boulder condition is replaced by the bulk velocity, *v*_*bulk*_.

**Fig. 7:**
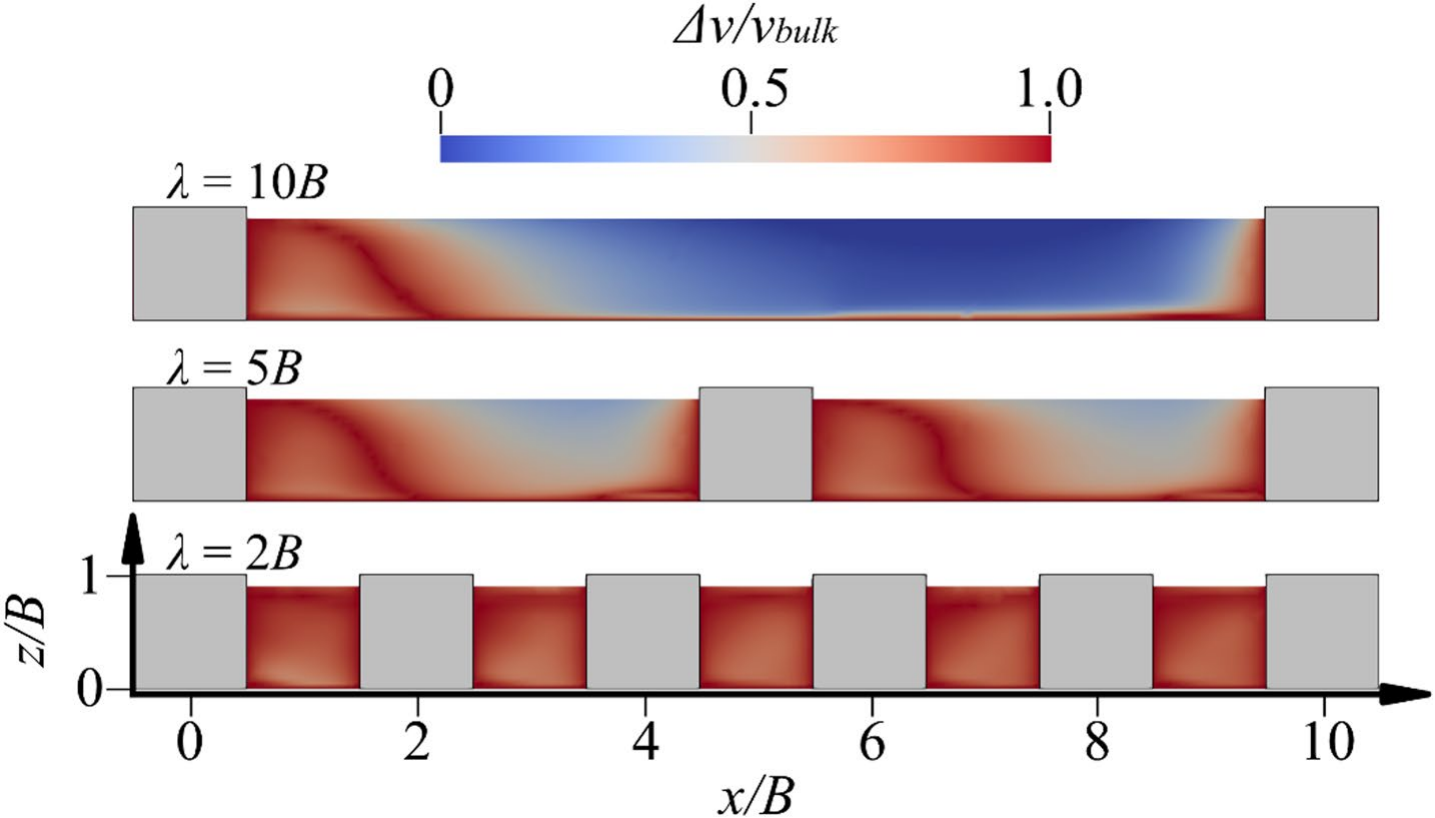
3D streamlines downstream of the boulder: (**a**) *λ* = 10*B*; (**b**) *λ* = 5*B*; (**c**) *λ* = 2*B*.

Figure 7 presents the near-bed flow reduction profiles at the centerplane *y* = 0. For *λ* = 10*B*, the velocity reduction is observed from the immediately downstream of the boulder to *x* = 3*B*, and this fits well the boundary layer recovery as shown in Fig. 6. From *x* = 3*B* to 9*B*, the value of *Δv* is small, and therefore, the boulder has no impact on the near-bed streamwise velocity implying that the boundary layer has almost fully recovered. The peak value of *Δv* is approximately 0.98*v*_*bulk*_ and observed at *x* = 1.5*B*. Dey et al.^11^ and Papanicolaou et al.^14^ have suggested a near-wake zone and a far-wake zone downstream of the spheres. The near-wake zone is defined as the zone where the reverse flow is observed, and the far-wake zone is defined as the zone where there is deceleration near the bed^27^. Based on these definitions, the total weak zone length is almost 4*B* at *λ* = 10*B*. For *λ* = 5*B*, a velocity reduction is observed at almost near bed regions between the boulders. From *x* = 0.5*B* to 3*B*, the deficit is because of the wake of the upstream boulder, whereas between *x* = 3*B* and 5*B*, the reduction is induced by the flow approaching the next boulder. For *λ* = 2*B*, the velocity reduction is clearly observed in all locations between the boulders. The wake zone and flow recovery are disturbed continuously by downstream boulders.

**Fig. 8:**
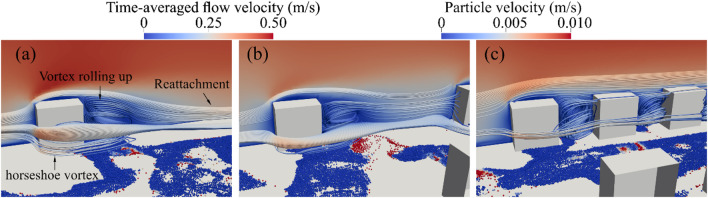
Near-bed flow reduction profiles along the centerplane at *y* = 0.

Additionally, Fig. 8 shows a time-averaged flow velocity field in the plane at *y* = 0 together with 3D streamlines. As the boulder-to-boulder space narrows, the character of the wake zone shifts from nearly isolated obstacle wakes to a merged recirculation zone. For *λ* = 10*B*, each block behaves much like an isolated obstacle. The streamline rolls up and reattach to the bed far downstream, while a distinct horseshoe vortex coils around the boulder’s base. As *λ* is reduced to 5*B*, the streamline structure around the boulder is similar to that at *λ* = 10*B*. The horseshoe vortex around the boulder induces a group of particle motion in the local region covered by the vortex in Fig. 8a and b but the streamlines leaving one boulder to impinge on the nose of the next before it can recover, leading to the interaction of two wake zones. At the most compact arrangement *λ* = 2*B*, the streamlines show an appreciable difference compared to those at the larger spacing. The recirculation vortex of the simulation is significantly smaller than that in the larger spacing simulations and the horseshoe vortex is weakened. Thus, decreasing the spacing transforms the flow from a series of isolated wakes to a low-momentum recirculation zone, thereby weakening vortex coherence while enhancing overall flow stagnation within the array.

### Sediment transport and depositions

**Fig. 9:**
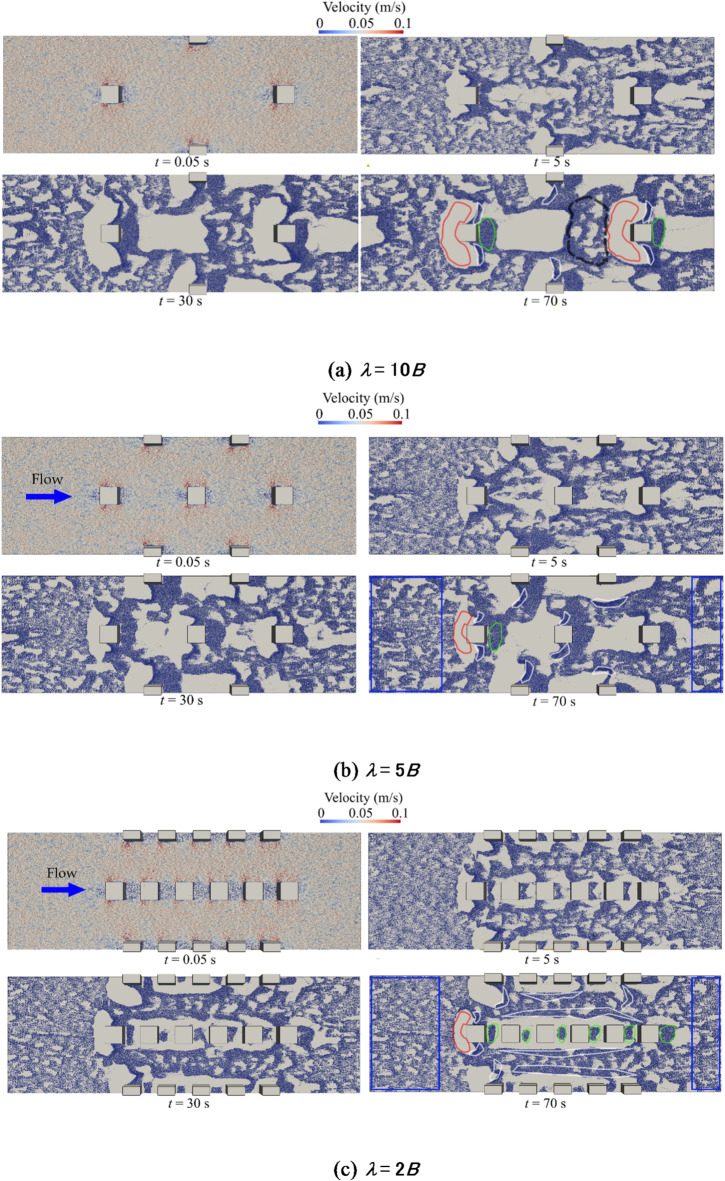
Process of sediment transport and deposition coloured by particle velocity.

Figure 9 illustrates the sediment transport and deposition processes under varying boulder concentrations. At *t* = 0.05 s, particles in all three scenarios are rapidly accelerated by the surrounding water flow. Subsequently, the particles are transported toward the outlet and re-enter through the inlet due to the periodic boundary condition applied at both ends (as shown in Fig. 5).

Sediment located immediately upstream of the boulders (highlighted in red in Fig. 9) is entrained due to the formation of near-bed horseshoe vortices at the boulder base. These coherent flow structures locally increase flow velocity and enhance near-bed shear stress, as shown in Fig. 8, thereby inducing sediment movement toward the lateral sides of the boulders. Downstream of the boulders, wakes are formed as a result of recirculation zones. Due to this flow recirculation, sediment is transported into the wake regions (indicated in green in Fig. 9), where it ultimately deposits.

As sediment continues to move toward the outlet, accumulation of particles occurs (shown in white in Fig. 9). This accumulation results from inter-particle contacts and the establishment of repose angles, which can increase resistance to particle motion^56,57^.

Distinct differences in sediment deposition patterns between boulders are observed across the three cases. For boulder spacings of *λ* = 5*B* and 10*B*, the green deposition regions maintain a similar extent. At *λ* = 10*B*, sediment deposition is also observed in the black dashed region upstream of the neighbouring boulder, a feature absent at *λ* = 5*B* due to the increased hydrodynamic interaction between boulders. Moreover, Fig. 6b shows the streamlines leaving one boulder to impinge on the nose of the next before it can recover, leading to the interaction of two wake zones at *λ* = 5*B*. However, at *λ* = 2*B*, the extent of the deposition region is significantly influenced by neighbouring boulders. As boulder concentration increases, sediment accumulation becomes more pronounced in the corridors between boulders, resulting in an overall increase in the size of the deposition regions within these areas. Moreover, sediment deposition pattern in the blue zones in Fig. 9 does not change considerably at *λ* = 2*B*. This implies that the flow’s sediment-entrainment capacity diminishes at higher boulder concentration.

**Fig. 10:**
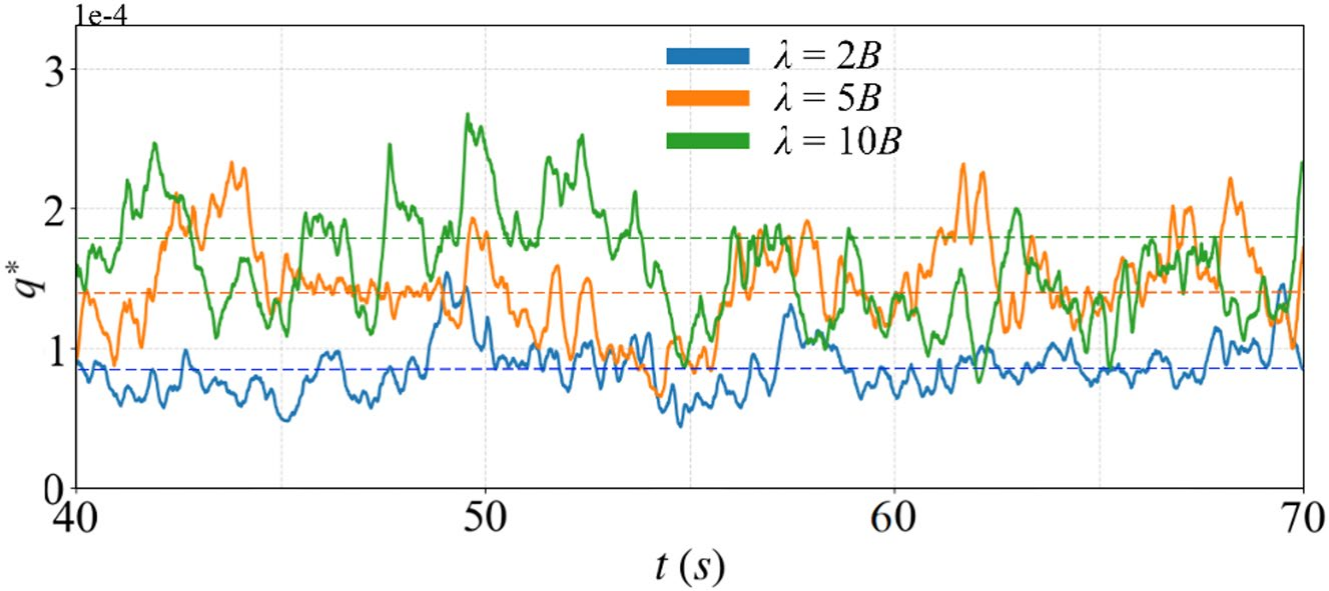
Non-dimensional sediment transport rate, $\:{q}^{*}$.

Figure 10 presents the non-dimensional sediment transport rate, $\:{q}^{*}$, for each case, as calculated using Eq. (15). The total bed area excluding the area occupied by boulders are used to calculate $\:{q}^{*}$. The time-averaged value of $\:{q}^{*}$ is calculated over the time from *t* = 40 s to 70 s. For *λ* = 10*B* and 5*B*, the time-averaged values of $\:{q}^{*}$are nearly identical, with $\:{q}^{*}$equal to 1.7 × 10^− 4^ and 1.5 × 10^− 4^, respectively. However, as the boulder concentration increases, the time-averaged values of $\:{q}^{*}$ decreases to 0.9 × 10^− 4^ at *λ* = 2*B*. This reduction is attributed to the significant weakening of turbulent vortices due to neighbouring boulders, which in turn diminishes the local sediment transport compared to cases with larger spacing, as illustrated in Figs. 8 and 9. Consequently, the amplitude of $\:{q}^{*}$ fluctuations at *λ* = 10*B* and 5*B* is greater than that observed at *λ* = 2*B*.

### Near-Bed shear stress

**Fig. 11:**
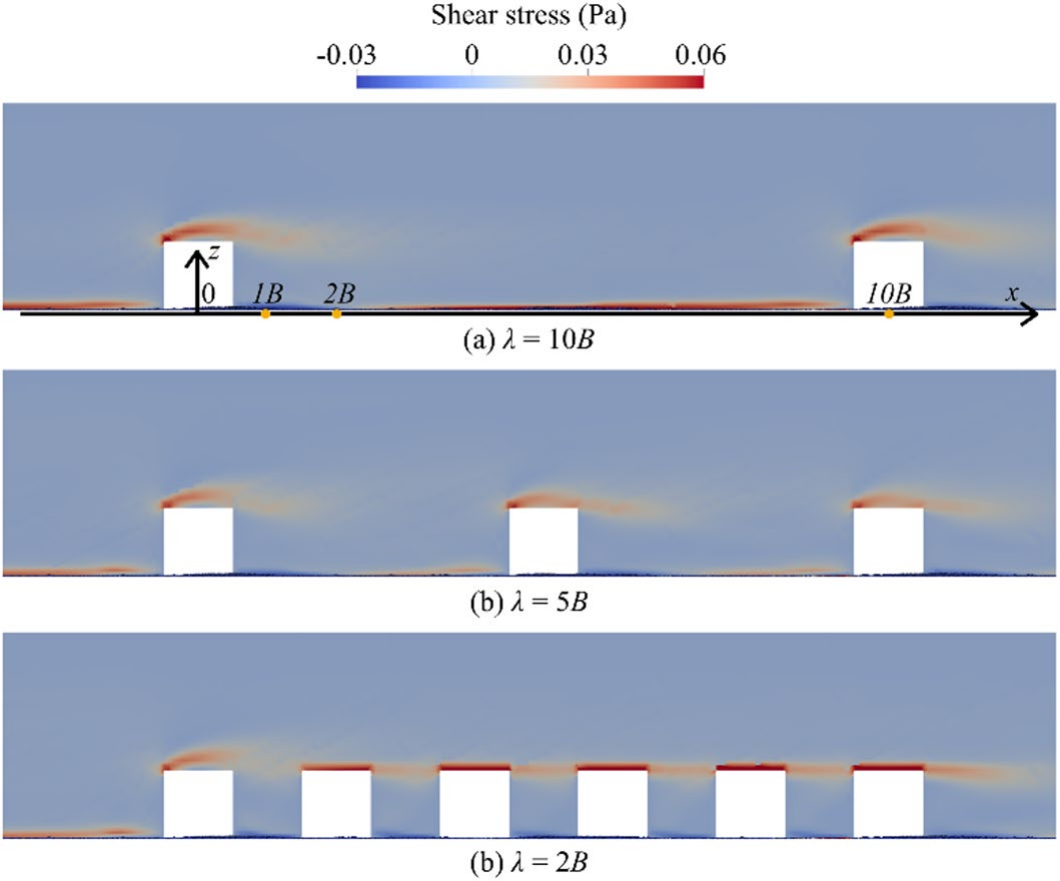
Distribution of shear stress, $\:{\tau\:}_{w}$, in the centre plane.

The shear stress, $\:{\tau\:}_{w}$, acting on the sediment bed be calculated with the following Eq. 

16 $$\:{\tau\:}_{w}={\mu\:}_{f}\frac{\partial\:{v}_{w}}{\partial\:z}$$

where $\:\frac{\partial\:{v}_{w}}{\partial\:z}$ is the streamwise water velocity gradient perpendicular to the direction of flow motion. Based on Eq. (16), Fig. 11 shows the $\:{\tau\:}_{w}$ fields at the centre plane (*y* = 0) of three simulation cases at *t* = 70 s. A region of high shear stress generated by flow separation at the boulder crest is observed. The bed shear stress upstream the boulder located at *x* = 0 gradually reduces with increasing boulder concentration. Moreover, the value of $\:{\tau\:}_{w}$ near the bed between boulders is significantly reduced as boulder concentration increases.

For $\:\lambda\:=$ 10*B*, the value of $\:{\tau\:}_{w}$ is low at the regions immediately upstream and downstream of the boulders. The increase of value of $\:{\tau\:}_{w}$ is observed along the streamwise direction downstream the boulder from *x* = 2*B* to 10*B* in Fig. 9a. Due to the isolated flow condition, the $\:{\tau\:}_{w}$ in the region upstream of the boulder located at *x* = 10*B* is nearly identical to that observed upstream of the boulder at *x* = 0*B*. For $\:\lambda\:=$ 5*B*, the profile of $\:{\tau\:}_{w}$ near the boulders is similar to the profile of the largest spacing but the values of $\:{\tau\:}_{w}$ upstream of neighbouring boulders are lower. This reduction is due to the influence of the adjacent boulder on the weak flow regime, as illustrated in Fig. 8b. For $\:\lambda\:=$ 2*B*, the values of $\:{\tau\:}_{w}$ between boulders are all negative because of the reverse flow shown in Fig. 8c.

## Discussions

### Sediment transport dynamics

The numerical simulations presented here reveal substantial effects of boulder concentration on sediment transport dynamics. At *λ* = 10*B* and 5*B* (isolated and wake interference regimes), sediment transport is mainly influenced by localized wake structures and horseshoe vortices around individual boulders, consistent with Yager et al.^10^, who observed reduced sediment mobility due to protection from large, immobile grains. Similarly, Papanicolaou et al.^27,58^ emphasized reductions in sediment transport driven by alterations in local shear stress distributions due to boulder-induced drag structures and turbulent eddies.

In this study, results show that increased boulder concentration substantially reduces sediment transport rates. In contrast, Fang et al.^15^ observed the opposite effect, reporting increased sediment transport rates with reduced boulder spacing. The contrast likely stems from differences in modelling approaches, sediment parameters, and flow conditions. Fang et al. performed LES simulations focusing primarily on turbulence features and local shear stress variations, whereas this study employs a coupled CFD–DEM approach to directly simulate interactions between fluid flow and sediment particles. Accordingly, sediment particle interactions such as accumulations and inter-particle contacts are visualized and modelled in our simulations. These particle interactions result in sediment accumulations and the establishment of repose angles between particles observed in Figs. 8 and 9, increasing the resistance to sediment motion^54,55^. The particle-fluid dynamics and sediment interactions may thus lead to differing results regarding the interplay between turbulence intensity, form drag, and sediment mobility. Additionally, variations in boulder geometries between the two studies could further explain this divergence in observed trends.

### Sediment deposition patterns

At large boulder spacing (isolated regime), the simulations reveal two primary depositional patterns: distinct wake patches and flank accumulation, as shown in Fig. 9a. These results are consistent with the high relative-submergence regime (HRS) described by Papanicolaou et al.^27^, where sediment preferentially accumulates in the wake. In contrast, earlier experiments with spherical boulders under low relative-submergence conditions (LRS)^19^ reported upstream sand ridges and stoss-side deposits, with minimal retention in the wake. This divergence may be attributed to two reasons. First, the simulations in this study maintain a submergence ratio of *H/B* = 3.5, within the HRS range favouring wake-zone entrapment. Second, the cubic geometry of the boulders in the simulations enhances flow separation and supports a broader, more coherent wake structure than spheres, promoting lee-side deposition. In contrast, spherical clasts under LRS exhibit smoother separation and generate necklace vortices that direct sediment toward stoss and flank zones.

With reduced spacing between cubes, depositional patterns shift from isolated wake patches to elongated sediment belts within inter-boulder corridors, as shown in Figs. 8c and 9c. Although not observed in sphere-based studies, this behaviour aligns with findings by Yager et al.^10^, who reported that high clast densities redistribute shear stress and encourage patch coalescence. Simulations show that closely spaced cubes create overlapping recirculation zones and reduced near-bed velocities, as shown in Fig. 7c, enabling stable sediment accumulation in corridor regions.

## Conclusions

This study investigates the effects of varying boulder concentration on sediment transport and deposition patterns using a coupled CFD–DEM approach. Three distinct flow regimes—isolated (*λ* = 10*B*), wake interference (*λ* = 5*B*), and skimming flow (*λ* = 2*B*)—were simulated, allowing a detailed analysis of the interplay between boulder spacing, local flow structures, sediment transport dynamics, and depositional characteristics.

The results indicate that increasing boulder concentration significantly alters local flow fields. As spacing decreases, discrete wakes behind isolated boulders transition into extensive recirculation zones spanning multiple boulders, leading to reduced turbulence intensity and weakened vortex structures. This flow transition notably reduces near-bed shear stresses, subsequently diminishing sediment transport rates, particularly at the highest boulder concentration (*λ* = 2*B*).

Sediment deposition patterns also show significant dependency on boulder concentration. In the isolated and wake interference regimes (*λ* = 10*B* and *λ* = 5*B*), sediment deposition occurs primarily in localized wake zones behind individual boulders. However, at higher boulder concentrations (*λ* = 2*B*), sediment deposition becomes pronounced within sheltered corridors formed between closely spaced boulders, creating elongated depositional belts.

This study provides insights into how boulder concentration affects sediment transport and deposition, providing the practical insights for boulder-related river restoration and habitat design. Future research may extend this work by incorporating natural boulder geometries, sediment heterogeneity, different submerged ratio and vegetation effects to better simulate natural river conditions.

## Data Availability

The data of the current study are available from the corresponding author on reasonable request.
